# Testis with no tunica vaginalis: A case report and literature review

**DOI:** 10.18502/ijrm.v19i9.9718

**Published:** 2021-10-10

**Authors:** Seyed Ali Mohamad Mirjalili, Hadi Maleki, Javid Gholami

**Affiliations:** ^1^Department of Urology, Shahid Sadoughi University of Medical Sciences, Yazd, Iran.; ^2^Department of Urology, Shahid Madani Hospital, School of Medicine, Alborz University of Medical Sciences, Karaj, Iran.; ^3^Urologist Gonbad Kavus Hospital, Golestan University of Medical Science, Gorgan, Iran.

##  Dear Editor

The testis and epididymis are surrounded by two layers of peritoneum called the Tunica vaginalis, which extends into the scrotum during the second and third embryonic months and has smooth muscle cells that aid spermatozoa movement (1). It is mesothelium tissue which includes visceral and parietal layers with an approximately 2-ml serosal fluid between the two layers (2). This fascial structure normally covers the entire testis except for the posterior surface. The visceral layer is an inner layer located on the tunica albuginea and the parietal layer is located on the extension of the scrotum.

During the second or third months of pregnancy, a vaginalis process derived from peritoneum in the abdomen goes into the scrotum. In the 7${}^{\mathrm{th}}$ to 9${}^{\mathrm{th}}$ months of pregnancy, the testes descend to the scrotum from the abdomen through this vaginalis process. The tunica vaginalis has smooth muscle cells that aim spermatozoa movement toward the testes and epididymis. The different layers of the tunica vaginalis and the space between the layers can be affected by several conditions such as congenital disorders, infection, inflammation, trauma, and neoplastic disorders (3, 4).

One of the most important components of the process of testes descending from the abdomen to the scrotum is the extending of the vaginalis process from the abdomen to the testes. In this case study, however, even though there was no history of abdominal surgery or scrotal disease and both testes were the same size, there was no tunica vaginalis in only one side.

At the Reproductive Medicine Research Institute, a 27-yr-old man (155 cm tall and weighing 47 kg) was diagnosed with azoospermia for his infertility. During the investigation, the karyotype, hormonal assay and chromosome Y micro-deletion were found to be normal. There was no history of any surgery (abdomen, testes), taking of medicine, or health problems (no past medical history). Upon physical examination, both testes were found to be the same size (volume) and smaller than normal (13 cc). He was evaluated for infertility and was a candidate for microtese due to azoospermia, where we have noticed there was no tunica vaginalis in right side while the left side was normal. The pathology report of his microtese stated maturation arrest.

Inguinal hernias and testicular and cord hydrocele are common congenital abnormalities known to be associated with Tunica vaginalis. The incidence of open vaginal processes in neonates is about 20% and most of these are asymptomatic (5, 6).

This condition has not been reported previously. The common pathology of tunica vaginalis includes cysts and mesothelioma (7, 8). Yu and colleagues reported an associated fibrous pseudo tumor, which has also been reported in other studies (9). Romano and Gonzalez-Serrano reported observing a partial agenesis of tunica albuginea (10).

In addition to physical examination, ultrasound is the most commonly available tool used for scrotal evaluation and it can play an important role in identifying the anatomy and distinguishing the pathologies of vaginalis processes. In this case, ultrasound may have played an important role in the diagnosis of tunica vaginalis agenesia before surgery.

Perhaps the most important limitation of this case report was the lack of ultrasonography of the scrotum before surgery.

Our findings indicate that during the second and the third months of pregnancy the peritoneal-derived vaginal process may not have been located in the scrotum on the right side. Also, the testis may descend from behind the peritoneum and the testis was less likely to pass through the vaginal process into the scrotum.

The absence of the tunica vaginalis does not seem to have had a significant impact on prognosis. However, there are no reports of drugs or surgical treatments in the literature to date. Whether or not the presence of a tunica vaginalis directly affects male fertility requires further research. The absence of the testicular tunica vaginalis has not been reported previously. However, this case report might be the descending of testis from the posterior of peritoneum or the testis moved to scrotum not through vaginal process.

Oral consent was obtained from participants for publishing this article.
